# Supplementary material for: Patterns of Intron Gain and Loss in Fungi
Source: PLoS Biol. 2004 Nov 30;2(12):e422. doi: 10.1371/journal.pbio.0020422 (PMC532390; doi:10.1371/journal.pbio.0020422)
Supplement: Table S1 — Also available at http://genes.mit.edu/NielsenEtAl/. (4.3 MB ZIP). [file pbio.0020422.st001.zip › NielsenEtAl/html/1374.html]

AN4683.1.NCU00669.1.MG02821.1.FG00189.1


```
 CLUSTAL W (1.82) Multiple Sequence Alignments - Introns Inserted


Sequence 1: MG02821.1	466 aa
Sequence 2: FG00189.1	458 aa
Sequence 3: NCU00669.1	466 aa
Sequence 4: AN4683.1	460 aa
Alignment Length: 472 aa
Number Identitical Residues: 168 aa
Alignment Score (without introns) 9325


MG02821.1 	MFKPILSLLFLLLAATVSAVSSSGNRLLAVLEDVAEKEKYSKFMGDLQS1RGFSITYSTP
NCU00669.1	-MRTLLSFLLLALASAVAAISTSGNRLLVVLDDVADKANYSKFFADLEE1REFKITYETP
FG00189.1 	-MRSLLSLVLFLFAALVSAVSTAGNRMLVVTDSPADKEAYTTFFRDLSE1RGYQITFESP
AN4683.1  	-MRWCSSLLLFGFLAAVNALSSSGSRLLVVLEDATEKGLYSTLWGDLEG1RGYNLIFESP
          	 ::   *:::: : : * *:*::*.*:*.* :. ::*  *:.:  **.  * :.: :.:*

MG02821.1 	KSDSLKLSHLGERVFDHVIIFPTKSKG1LGPNLTPALLLQFVKDEGNVMLALSAETAVPI
NCU00669.1	KSDSLTLFSLGERTYDHVLFLPVRTKG~LGPNLTPKTLHDFVNAKGNILLALSANTTTPT
FG00189.1 	KTESLALFLHGERTYDHLVFLPTKIKG1LGPNLTPQLILEFVTAGGNLLVTMSSNHVVPS
AN4683.1  	KNEQLSLFELGERAYDHVLLLPPKSKG1FGPSLSPKNLVDFLNSEGNILLALSGKSTTPS
          	*.:.* *   ***.:**::::* : ** :**.*:*  : :*:.  **:::::*.: ..* 

MG02821.1 	SIVSLLHELDIQLPADRTGLVVDHFHYDKISAAEKHDVILVK-PRPVRDFHKDIFGSEKQ
NCU00669.1	SLIALLSELDITLPTDRNSLVVDHFNYDITSATDKHDVLLLPPPKPTRAGIKDFFSEGAG
FG00189.1 	ALDAALMELEIMVPEERTGKVVDHFSYDTISASEDHDVLVVEAPRNILPGRKDYFEIP--
AN4683.1  	AVSSLLLEFDLHLSTDRSSITVDHFNYDTLSASEKHDVLLLERPGKLRYDTKEFFSGE--
          	:: : * *::: :. :*.. .**** **  **::.***:::  *       *: *     

MG02821.1 	TSQLLAVPRAIGQTLGQSPLLNPVLRAPLTAYSYDPKEQSE--VVDDLFAAGEQLSLVSA
NCU00669.1	-TKLIAFPRGVGAVLGNGQLLNPILRAPRTAYSYNPKEQVEAVNGDDLFAAGQQLSLVST
FG00189.1 	-GAFLSVPHALGHRLGNGELITPVLRAPPTAYIYNPKDQNKTIETDDLFAAGTQVNLVTV
AN4683.1  	--GVVAVPSASPHTLGDNSLLAPILRAPATAYSYNPKEDTG--SVEDVFATGSQLALVSA
          	   .::.* .    **:. *: *:**** *** *:**::      :*:**:* *: **:.

MG02821.1 	VQPRNSARVTVVGSAEMLQDQWFDAEVKPSSSNKKVSTYNREFAKKLSGWTFQEYGVLRV
NCU00669.1	MQARNNARVTVVGSAEMLQDKWFDASVTKLDGQPVVQTFNREFAKRVSGWTFQEIGVLKV
FG00189.1 	FQARNSARITVIGSAEMLQDKTFDTKVARQGG-KPQFPANREFVTNVAAWTFQELGVLRV
AN4683.1  	MQARNSARFTVLGSVETLQDKWFSATVKAPGG-EEVQTVNREFAKQLTAWTFKETGVLKV
          	.*.**.**.**:**.* ***: *.: *   ..     . ****...::.***:* ***:*

MG02821.1 	NSVEHHLNEAGA-SNVSNPAIYRVKNDV0TYTISLSEYKPETQSWGPFTVPDGDEIQLEF
NCU00669.1	NWVEHHLEEEGA-PNDSNPEMYRVKNNV0SYTISLSEFAWD--HWIGFNVPEHDEIQLEF
FG00189.1 	NSIEHRLDGS----NETNPAGYRVKNDV0SYNMSLSEYAWN--TWQPYQTPEDDEIQLEF
AN4683.1  	GKIEHHLAKDDLTAEDLNPSIYRIKNET0VFSIEISEYNYD--GYVPFEVPVNDNIQLEF
          	. :**:*   . :.:  **  **:**:.  :.:.:**:  :   :  : .*  *:*****

MG02821.1 	SMLSPFHRLGLKPTTSTESDATKYSVSFTLPDQHGIFNFRVNYKRPFLTYIDSKDQVSVR
NCU00669.1	SMLSPFHRLNLK-EESHRLAAVHYHTVFTTPDQHGIFNFMVNYKRPFLSHIEEKNTVPVR
FG00189.1 	TMLSPFWRLPLKPILSND-HETIYSTNFTLPDQHGIFNFKVNYKRAFLTYIEKKNTVSVR
AN4683.1  	TMLSPFHRLNLQQSATTP-NSTIFSTRFTVPDQHGIFSFRVNYKRPFLTNIEEKHEVTVR
          	:***** ** *:   :     . : . ** *******.* *****.**: *:.*. *.**

MG02821.1 	HMAHDEWPRSFVISGAWPWITGIGATITGFLAFCLVFMFSAPT--GKATVTKKTQ
NCU00669.1	HMAHNEWPRSYVISGAWPWIAGLGSTVVGFLGFVALWMYSAPVDAGKKVTVKKTQ
FG00189.1 	HLAHDEYDRSYSINGAWTGIAGTWATIIGFISFCFIWLYSKPV--PKTFLDKKTQ
AN4683.1  	HFAHNEYPRSWKITGGWVWIAGLWSVIVGFLAFVVVWLYSEPAT-AEKAKTKKTQ
          	*:**:*: **: *.*.*  *:*  :.: **:.*  ::::* *.   :    ****
```
